# Signals Alongside Scans: A Genomics-Guided Framework for Liquid Biopsy in Bone and Soft-Tissue Sarcomas

**DOI:** 10.3390/cells15141271

**Published:** 2026-07-15

**Authors:** Ibrahim Alabid, Ali Jad Yousef, Mohamedanas Mohamedfaruk Patni, Radwan Abdulaziz Aloti, Zain Al-Abdeen Mohammed Qassim, Ayman Ahmad Alothman-Agha, Hesham Amin Hamdy, Feras Mohammed Noury, Mohamed Tarek Abdelfattah

**Affiliations:** 1 MBBS, RAK College of Medical Sciences, Ras Al Khaimah Medical and Health Sciences University (RAKMHSU), Ras Al Khaimah 11172, United Arab Emirates; radwan.21901025@rakmhsu.ac.ae (R.A.A.); zain.21901086@rakmhsu.ac.ae (Z.A.-A.M.Q.); ayman.21901015@rakmhsu.ac.ae (A.A.A.-A.); mohamed.21901027@rakmhsu.ac.ae (M.T.A.); 2 Department of Surgery, RAK College of Medical Sciences, RAK Medical and Health Sciences University, Ras Al Khaimah 11172, United Arab Emirates; alijad@rakmhsu.ac.ae (A.J.Y.); hesham@rakmhsu.ac.ae (H.A.H.); 3 Department of Community Medicine, RAK College of Medical Sciences, Ras Al Khaimah Medical and Health Sciences University, Ras Al Khaimah 11172, United Arab Emirates; 4 Consultant Orthopaedic and Joint Replacement Surgeon, Department of Spine and Orthopaedic Surgery, RAK Hospital, Ras Al Khaimah 11393, United Arab Emirates; dr_noury@hotmail.com

**Keywords:** liquid biopsy, circulating tumor DNA, circulating tumor cells, bone sarcoma, soft-tissue sarcoma, osteosarcoma, minimal residual disease, molecular relapse, genomic surveillance

## Abstract

**Highlights:**

**What are the main findings?**

- Liquid biopsy in bone and soft-tissue sarcomas is most clinically meaningful when assay selection is guided by tumor biology, molecular architecture, and the clinical question being addressed.
- ctDNA currently has the most mature evidence base among liquid-biopsy analytes in sarcoma, particularly for treatment-response monitoring, postoperative minimal residual disease assessment, and molecular relapse detection in osteosarcoma and translocation-associated sarcomas.

**What are the implications of the main findings?**

- A genomics-guided framework can help match sarcoma subtypes to appropriate liquid-biopsy strategies, including breakpoint-guided, tumor-informed, mutation-based, methylation-based, fragmentomic, and copy-number-based assays.
- Liquid biopsy should currently be integrated as an adjunct to imaging and multidisciplinary surveillance, rather than replacing standard radiologic follow-up, until prospective studies define clinical actionability, sampling schedules, thresholds, and outcome benefit.

**Abstract:**

Background: Bone and soft-tissue sarcomas are rare, heterogeneous malignancies whose surveillance remains dominated by imaging despite substantial molecular diversity and variable patterns of relapse. Circulating tumor DNA (ctDNA) and circulating tumor cells (CTCs) offer minimally invasive approaches for monitoring tumor biology, but their performance in sarcoma depends strongly on subtype, disease burden, assay design, and biological shedding. Methods: This narrative review synthesizes evidence published from 2015 to 2026 on ctDNA and CTCs for baseline risk assessment, treatment-response monitoring, minimal residual disease (MRD) detection, molecular relapse, and integration with imaging-based surveillance in bone and soft-tissue sarcomas. Results: Current evidence supports a genomics-guided framework in which liquid-biopsy strategy is selected according to sarcoma subtype, molecular architecture, and clinical purpose. ctDNA is the most mature analyte, with best-supported evidence in osteosarcoma, where tumor-informed assays predict postoperative relapse, and in translocation-associated sarcomas, where breakpoint-guided assays enable highly specific longitudinal monitoring. Copy-number-based approaches are relevant for complex-karyotype tumors, while mutation-, methylation-, fragmentomic-, and RNA-based strategies may be useful in selected contexts. However, detection rates vary, false-negative results occur in low-shedding or low-volume disease, and clinical utility for changing treatment remains incompletely established. CTCs provide complementary cellular and prognostic information, particularly in osteosarcoma, but remain limited by platform heterogeneity and incomplete standardization. Conclusion: Liquid biopsy may refine risk stratification, support treatment-response assessment, clarify indeterminate imaging findings, and identify molecular relapse in selected sarcoma patients. At present, it should be interpreted as an adjunct to imaging and specialist multidisciplinary care rather than as a replacement for standard radiologic surveillance.

## 1. Introduction

Bone and soft-tissue sarcomas are rare, heterogeneous mesenchymal malignancies that remain difficult to monitor across diagnosis, treatment, and follow-up [1,2]. Their diversity extends beyond histology to include distinct molecular architectures, ranging from translocation-associated tumors with relatively simple genomic profiles to complex-karyotype sarcomas characterized by widespread copy-number alterations, structural instability, methylation changes, and substantial intertumoral heterogeneity [3,4]. This biological variation is a major reason why biomarker development in sarcoma has progressed more slowly than in common epithelial malignancies [5]. Current sarcoma surveillance remains centered on imaging and clinicopathologic assessment. These tools are essential and should remain foundational; however, they mainly capture anatomic disease rather than real-time molecular activity [6,7,8]. In the postoperative and post-treatment setting, radiologic interpretation may be further complicated by fibrosis, necrosis, inflammation, postsurgical change, and stable residual abnormalities that do not reliably distinguish treatment effect from viable disease [6,7,8]. Conversely, clinically relevant recurrence may be biologically active before it becomes radiographically measurable.

Liquid biopsy has already transformed several areas of oncology, particularly advanced-cancer genotyping and treatment-resistance monitoring. In early-stage solid tumors, ctDNA detection after curative-intent therapy has shown strong clinical validity for predicting future relapse, although its clinical utility for directing treatment escalation or de-escalation remains under prospective evaluation [9,10]. Importantly, many limitations of ctDNA analysis are not unique to sarcoma. Low circulating tumor fraction, false-negative plasma results, sampling timing, clonal hematopoiesis, assay sensitivity, pre-analytical variability, and biological differences in tumor shedding affect ctDNA interpretation across solid tumors [11,12]. What makes sarcoma particularly challenging is the combination of rarity, marked histologic and genomic heterogeneity, variable tumor shedding, low-volume localized relapse, and the absence of a single recurrent genomic alteration across most subtypes.

Among liquid-biopsy analytes, ctDNA is currently the most clinically mature for sarcoma monitoring because it can capture tumor-specific genomic and epigenomic alterations through a minimally invasive and repeatable blood test [5,9]. However, sarcoma should not be approached as a single liquid-biopsy entity. In translocation-associated tumors such as Ewing sarcoma, synovial sarcoma, and myxoid liposarcoma, disease-defining structural variants can be tracked using breakpoint-guided or fusion-aware assays [13,14,15]. In complex-karyotype sarcomas such as osteosarcoma, undifferentiated pleomorphic sarcoma, leiomyosarcoma, and dedifferentiated liposarcoma, individualized tumor-informed approaches or genome-wide copy-number strategies may be more appropriate [16,17]. Similarly, mutation-based assays may be informative in selected chondrosarcoma cases, while methylation-based approaches may provide value in osteosarcoma, chordoma, and other low-mutation contexts [18,19,20,21].

The clinical evidence also supports subtype-specific interpretation. In Ewing sarcoma and other translocation-associated tumors, disease-defining rearrangements in plasma have enabled molecular diagnosis, longitudinal monitoring, and identification of progression in selected cohorts [13,22,23]. In synovial sarcoma and myxoid liposarcoma, subtype-matched structural-variant tracking further supports the need to align assay design with tumor biology [14,15,24]. In osteosarcoma, tumor-informed and copy-number-based approaches have shown value for relapse-risk stratification and treatment-response monitoring despite marked genomic heterogeneity [16,17,21].

CTCs represent a complementary but less mature biomarker modality. In osteosarcoma, CTC burden, phenotype, and dynamic changes have been associated with metastatic disease, treatment response, and prognosis [25,26,27,28]. In soft-tissue sarcomas, CTC detection has been shown to be feasible, but evidence remains limited by small cohorts, platform heterogeneity, and incomplete standardization [29]. Copy-number alterations should not be viewed only as a limitation for sarcoma ctDNA analysis. In complex-karyotype sarcomas, CNA-based plasma approaches, including low-pass or ultra-low-pass whole-genome sequencing, may provide useful information on tumor fraction and disease burden. Their main limitation is not biological irrelevance but reduced sensitivity in low-burden MRD settings and possible underperformance in fusion-driven tumors where the dominant signal is a structural breakpoint rather than broad CNA burden. Therefore, a rational sarcoma liquid-biopsy strategy must match the assay to the tumor’s molecular architecture and to the clinical question being asked.

In this review, we use the term “genomics-guided” to describe assay selection informed by the tumor’s known or expected molecular features. This does not mean replacing tissue diagnosis, histopathology, molecular pathology, or imaging. Rather, it refers to selecting the liquid-biopsy analyte and assay after considering fusion status, recurrent mutations, copy-number profile, methylation pattern, fragmentomic features, or patient-specific tumor variants. This review aims to place liquid biopsy in bone and soft-tissue sarcomas within the broader ctDNA field, summarize quantitative evidence for ctDNA and CTC performance across sarcoma subtypes, and propose a clinically practical framework for integrating liquid biopsy with imaging-based surveillance.

## 2. Literature Review Search Strategy

This narrative review synthesizes current evidence on circulating tumor DNA (ctDNA), cell-free DNA (cfDNA), cell-free RNA (cfRNA), circulating tumor cells (CTCs), and other circulating biomarkers as tools for baseline risk assessment, treatment-response monitoring, minimal residual disease (MRD) detection, molecular relapse surveillance, and integration with imaging-based follow-up in bone and soft-tissue sarcomas. The review emphasizes subtype biology, assay design, clinical applicability, implementation barriers, and evidence limitations.

A structured literature search was conducted in PubMed and Scopus for studies published between January 2015 and 1 April 2026. The final search was performed on 1 April 2026. The PubMed search strategy was as follows: (“sarcoma” OR “bone sarcoma” OR “soft tissue sarcoma” OR “osteosarcoma” OR “Ewing sarcoma” OR “chondrosarcoma” OR “chordoma” OR “synovial sarcoma” OR “myxoid liposarcoma” OR “rhabdomyosarcoma”) AND (“liquid biopsy” OR “circulating tumor DNA” OR “ctDNA” OR “cell-free DNA” OR “cfDNA” OR “cell-free RNA” OR “cfRNA” OR “circulating tumor cells” OR “CTCs” OR “exosome” OR “extracellular vesicle”) AND (“minimal residual disease” OR “MRD” OR “molecular relapse” OR “relapse” OR “surveillance” OR “monitoring” OR “treatment response” OR “prognosis” OR “molecular profiling”). The Scopus search used the same conceptual terms with TITLE-ABS-KEY fields.

Additional targeted searches were performed to identify systematic reviews, translational assay studies, imaging and surveillance guidance documents, clinical practice guidelines, technical consensus statements, and recent reviews relevant to ctDNA assay validation, CTC methodology, cfRNA, extracellular vesicles, radiomics, artificial intelligence, and clinical implementation. Reference lists of selected articles were also manually screened to identify additional relevant publications.

Records were screened in two stages. First, titles and abstracts were reviewed for relevance to sarcoma, liquid biopsy, circulating biomarkers, molecular monitoring, MRD, recurrence detection, treatment-response assessment, or surveillance. Second, full texts were assessed for relevance to clinical interpretation, assay strategy, subtype-specific application, or implementation. Peer-reviewed original research articles were prioritized, especially longitudinal clinical studies, translational biomarker investigations, prospective or retrospective cohort studies, and subtype-specific analyses. High-quality systematic reviews, narrative reviews, genomic characterization studies, imaging guidelines, assay-validation papers, and pre-analytical consensus documents were included when they provided essential biological, clinical, or methodological context. Studies were excluded if they were not directly relevant to sarcoma, liquid biopsy, circulating biomarkers, molecular disease monitoring, or clinical implementation. Articles focused exclusively on non-sarcoma malignancies were excluded unless they provided essential general ctDNA, MRD, assay-validation, or radiomics/artificial-intelligence context. Older landmark studies were included selectively when necessary to establish the biological or methodological foundations of sarcoma genomics or liquid-biopsy development.

**Figure 1:**
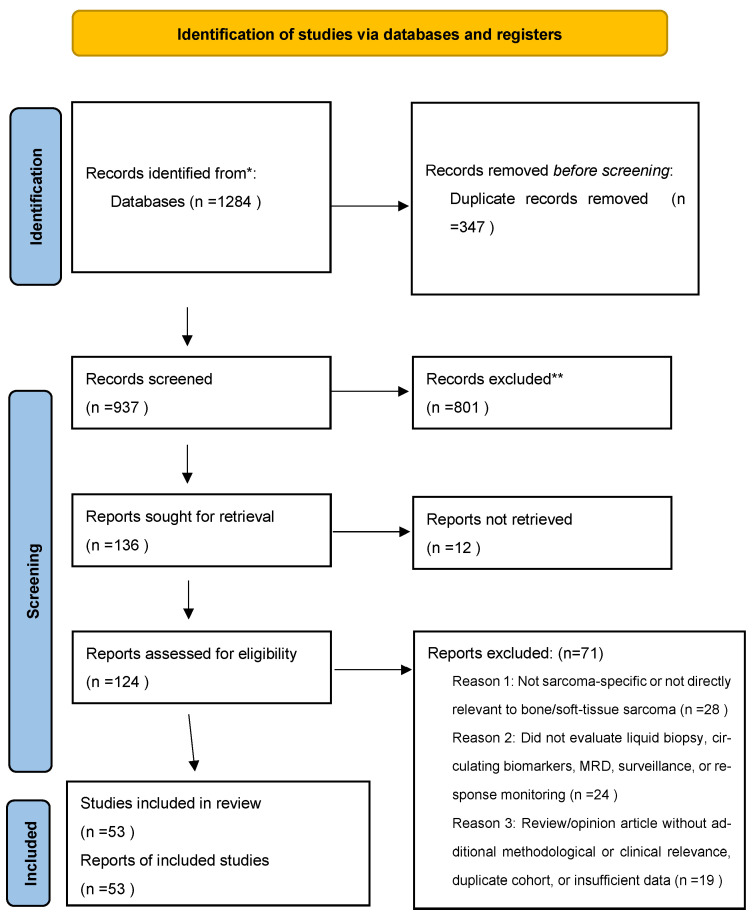
Study-selection workflow for the narrative review. The diagram summarizes database identification, duplicate removal, title and abstract screening, full-text assessment, and final source inclusion. Because this article is a narrative review rather than a formal systematic review or meta-analysis, the workflow is presented to improve methodological transparency rather than to indicate full PRISMA-based systematic-review methodology. * Identified from PubMed and Scopus. ** Records were excluded at title/abstract screening primarily because they were (1) not sarcoma-specific or not directly relevant to bone or soft-tissue sarcoma, or (2) not focused on liquid biopsy, circulating biomarkers, MRD, treatment-response monitoring, surveillance, or clinical implementation.

Because this article is a narrative review rather than a formal systematic review or meta-analysis, PRISMA 2020 methodology was not fully applied and no quantitative meta-analysis was performed. However, key PRISMA transparency principles were used to improve reproducibility of the search and selection process. The study-selection workflow is summarized in Figure 1. The database search identified 1284 records. After removal of 347 duplicates, 937 records underwent title and abstract screening. Of these, 801 records were excluded because they were not directly relevant to sarcoma liquid biopsy, circulating biomarkers, MRD, response monitoring, surveillance, or clinical implementation. Full texts of 136 reports were sought, of which 12 could not be retrieved. A total of 124 full-text reports were assessed for eligibility, and 71 were excluded because they were not sarcoma-specific, did not evaluate liquid biopsy or clinically relevant circulating biomarkers, or lacked sufficient clinical or methodological relevance. Finally, 53 sources were included in the narrative synthesis.

## 3. Sarcoma in the Broader ctDNA Landscape

ctDNA analysis has developed most rapidly in common epithelial malignancies, where it is now used for selected advanced-cancer genotyping scenarios and is being actively evaluated for MRD and molecular relapse monitoring after curative-intent therapy [5,9,10]. Across early-stage solid tumors, postoperative or post-treatment ctDNA detection is consistently associated with a higher risk of recurrence, but this prognostic validity should be distinguished from clinical utility. A biomarker may predict relapse without yet proving that biomarker-guided intervention improves survival. This distinction is especially important in sarcoma, where effective salvage strategies vary by subtype, disease site, relapse pattern, and prior treatment exposure.

The broader oncology literature also clarifies why assay choice matters. MRD detection requires greater analytical sensitivity than baseline genotyping or advanced-disease resistance monitoring, because tumor-derived DNA may represent only a very small fraction of total cell-free DNA after definitive therapy [5,9]. Tumor-informed assays can improve sensitivity by tracking patient-specific alterations identified from tissue sequencing, whereas tumor-naïve approaches may be useful when tissue is unavailable or when methylation, fragmentomic, or copy-number signals provide sufficient discriminatory power [9,10,11,12]. These principles apply to sarcoma but require adaptation because sarcoma subtypes differ profoundly in their dominant genomic signals.

Therefore, the central question in sarcoma is not whether liquid biopsy is useful in general, but which liquid-biopsy strategy is appropriate for a given subtype and clinical scenario. Fusion-driven tumors may be best suited to breakpoint-guided ddPCR or fusion-aware NGS [13,14,15]. Complex-karyotype tumors may require tumor-informed sequencing, CNA-based approaches, or methylation/fragmentomic profiling [16,17,18,19,20,21]. Mutation-defined subtypes may be suited to hotspot assays when the mutation is stable and tumor-specific [18,20]. This framework avoids the misleading assumption that one uniform liquid-biopsy platform can serve all sarcoma surveillance needs.

## 4. Biological Determinants of ctDNA Detectability in Sarcoma

ctDNA detectability is governed by both assay design and tumor biology. Across solid tumors, ctDNA fraction varies according to tumor burden, anatomical site, proliferation, apoptosis, necrosis, inflammation, treatment exposure, vascularity, microenvironment, and host-related factors [5]. These determinants are not sarcoma-specific, but their impact is amplified in sarcoma because clinically important events often occur in low-volume postoperative or localized settings where ctDNA shedding may be limited.

Several biological scenarios may produce false-negative or “not detected” ctDNA results despite viable disease. Small local recurrences, indolent tumors, poorly vascularized lesions, anatomically sequestered sites, low-volume pulmonary or soft-tissue disease, and post-treatment fibrosis may all release insufficient tumor DNA into plasma. Conversely, high tumor burden, metastatic disease, necrosis, rapid proliferation, and active progression may increase ctDNA release. These factors help explain why ctDNA detection is often more reliable in advanced or progressive disease than in early MRD settings.

This distinction has direct clinical implications. A positive ctDNA result may be highly informative when assay specificity is strong, especially when the tracked alteration is tumor-specific. In contrast, an undetectable result should be interpreted as “ctDNA not detected” rather than definitive absence of disease [5]. This language is particularly important in sarcoma surveillance, where low-shedding localized recurrences may remain below the limit of plasma detection. Liquid-biopsy interpretation should therefore incorporate tumor subtype, prior detectability, disease burden, treatment timing, imaging findings, and pretest probability of recurrence.

Pre-analytical variables further influence interpretation. Collection tube type, sample volume, time to processing, centrifugation protocol, storage conditions, freeze–thaw exposure, DNA extraction method, cfDNA input, sequencing depth, and error-suppression strategy can all affect assay performance [11,12]. In rare and low-shedding tumors such as sarcoma, these variables are not minor technical details but central determinants of whether a result is clinically interpretable.

## 5. Genomically Informed Assay Selection and Assay Foundations

**Figure 2:**
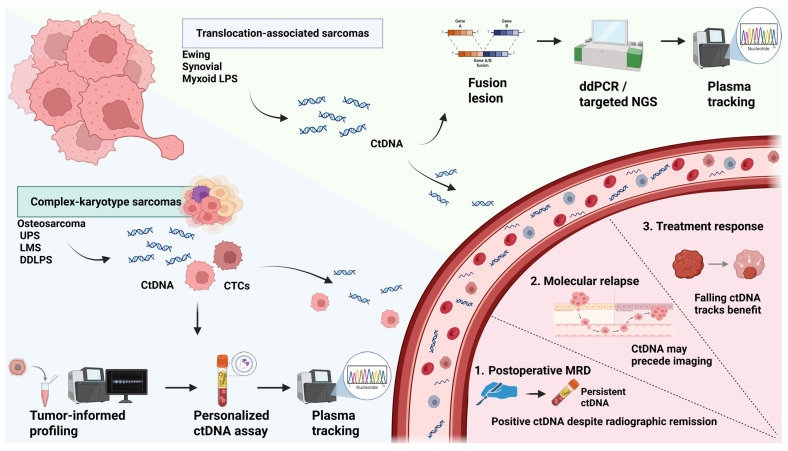
Genomics-guided liquid-biopsy framework in sarcoma. Standard tissue diagnosis, histopathology, molecular pathology, and imaging remain foundational. Liquid-biopsy assay selection is then guided by sarcoma subtype, molecular architecture, and the clinical question. Translocation-associated sarcomas are suited to fusion- or breakpoint-guided ctDNA assays, whereas complex-karyotype sarcomas may require tumor-informed, personalized, methylation-based, fragmentomic, or copy-number-based approaches. Liquid biopsy may support baseline risk assessment, treatment-response monitoring, postoperative MRD detection, and molecular relapse surveillance but should complement rather than replace imaging. Abbreviations: ctDNA, circulating tumor DNA; CTCs, circulating tumor cells; ddPCR, digital droplet PCR; NGS, next-generation sequencing; MRD, minimal residual disease; LPS, liposarcoma; UPS, undifferentiated pleomorphic sarcoma; LMS, leiomyosarcoma; DDLPS, dedifferentiated liposarcoma.

A genomically informed liquid-biopsy strategy is particularly important in sarcoma because these tumors do not share a uniform molecular architecture. Broadly, sarcomas can be divided into translocation-associated tumors with relatively simple genomes and complex-karyotype tumors characterized by widespread copy-number alterations, structural instability, and substantial intertumoral heterogeneity [3,4]. This distinction is operational rather than merely taxonomic: it determines which circulating analyte is informative, which assay design is feasible, and which clinical application is realistic. A plasma assay effective in a fusion-driven sarcoma may underperform in a copy-number-dominant tumor, whereas genome-wide CNA approaches may be less informative when the key disease signal is a specific breakpoint. This assay-selection logic is summarized in Figure 2.

Translocation-associated sarcomas provide the clearest illustration of assay-subtype matching. In Ewing sarcoma, synovial sarcoma, and myxoid liposarcoma, the disease-defining lesion is highly tumor-specific and can be tracked using breakpoint-directed ddPCR, targeted NGS, or fusion-aware sequencing approaches [13,14,15,22,23,24]. When a stable genomic breakpoint is known, ctDNA can function as a highly specific marker of active disease. However, sensitivity may remain limited in low-volume or localized recurrence, and assay design must account for breakpoint heterogeneity, cfDNA input, and longitudinal reproducibility [13,22,23,24].

Complex-karyotype sarcomas require a different strategy. Tumors such as osteosarcoma, undifferentiated pleomorphic sarcoma, leiomyosarcoma, myxofibrosarcoma, and dedifferentiated liposarcoma often lack a single recurrent alteration and instead exhibit heterogeneous combinations of copy-number changes, structural variants, methylation signatures, and patient-specific mutations [3,16,17,21]. In these settings, tumor-informed approaches are conceptually applicable because they do not depend on predefined recurrent targets. Their main limitations are practical rather than biological: adequate tumor tissue must be available, tumor purity must be sufficient, matched normal sequencing may be required, turnaround time and cost may be substantial, and clinical validation remains limited.

ddPCR remains the most efficient platform when the genomic target is known. This explains its utility in translocation-associated sarcomas, EWSR1 fusion-positive tumors, and selected mutation-driven tumors such as IDH-mutant chondrosarcoma [13,18,20,23]. ddPCR provides high sensitivity, rapid turnaround, and absolute quantification, but its limited multiplexing capacity restricts its use in genomically heterogeneous tumors where no single alteration is consistently informative.

Targeted NGS expands the analytical scope by enabling simultaneous interrogation of multiple alterations, including structural variants, hotspot mutations, and copy-number changes in selected platforms. In pediatric sarcomas, CAPP-Seq has demonstrated the feasibility of detecting both translocations and copy-number alterations from plasma, with ctDNA levels correlating with metastatic status, clinical response, and impending relapse [30]. These approaches are particularly useful when several tumor-defining or patient-specific events can be tracked simultaneously.

Baseline ctDNA detection may also provide prognostic information. In a Children’s Oncology Group cohort, ctDNA was detectable in more than half of newly diagnosed Ewing sarcoma and osteosarcoma cases, and detectable ctDNA was associated with inferior outcomes, particularly in localized Ewing sarcoma [31].

Broad commercial ctDNA panels may identify alterations in selected advanced soft-tissue sarcoma cases, but concordance with tissue profiling remains variable, particularly for copy-number events and tumors with low circulating tumor fractions [32]. This distinction is important because broad plasma profiling for advanced disease is not equivalent to highly sensitive MRD monitoring.

Low-pass or ultra-low-pass WGS occupies a selective but important role. Its main advantage is genome-wide CNA detection without requiring predefined mutation panels, making it conceptually attractive for complex-karyotype sarcomas [33]. Pilot data suggest that LP-WGS can detect ctDNA in a variety of sarcoma subtypes, but sensitivity varies and may be lower in translocation-associated tumors with limited CNA signal [33]. Therefore, CNA-based assays should be positioned as useful for tumor-fraction estimation and disease-burden assessment, rather than as universally sensitive MRD tools.

Large-panel ctDNA sequencing may further support precision profiling in advanced soft-tissue sarcoma, particularly when tissue biopsy is unavailable or when rapid molecular profiling is clinically relevant [34]. However, advanced-disease profiling should not be conflated with postoperative MRD detection, where tumor fraction is expected to be much lower and assay sensitivity requirements are higher.

In myxoid liposarcoma, combined detection of translocation breakpoints and selected SNVs may improve quantitative monitoring and specificity compared with single-signal approaches [35]. This example illustrates that even within translocation-associated sarcomas, assay refinement can improve clinical interpretability.

Mixed bone and soft-tissue sarcoma feasibility studies also support tumor-informed detection approaches, but the evidence remains preliminary and requires subtype-specific validation [36]. Similarly, plasma tumor DNA appears more reproducible than plasma tumor RNA in Ewing sarcoma, suggesting that cfRNA should remain investigational rather than being presented as equivalent to ctDNA for routine monitoring [37]. Mutation-based ctDNA detection in chordoma is promising but remains early and requires further validation [38].

CTC-based methodologies remain more heterogeneous. Surgical CTC detection in osteosarcoma provides proof-of-principle evidence, but these data should be interpreted as pilot-level support rather than as evidence for routine surveillance [39].

Across all platforms, pre-analytical and analytical rigor is essential. Blood collection tube type, sample volume, time from venipuncture to plasma separation, centrifugation protocol, storage temperature, freeze–thaw exposure, and cfDNA extraction method can all influence cfDNA yield, leukocyte DNA contamination, fragment size, tumor fraction, and downstream assay performance [5,11,12,40]. Analytical variables are equally important, including cfDNA input, sequencing depth, molecular barcoding, error-suppression strategy, limit of detection, limit of quantification, bioinformatic filtering, and handling of clonal hematopoiesis. For sarcoma studies, these details should be reported transparently because low tumor fraction and low-shedding disease may convert an otherwise valid assay into a non-informative result. Future studies should therefore report plasma volume, cfDNA input, tumor fraction when available, assay sensitivity, limit of detection, positivity threshold, sequencing depth, error-correction method, matched white-blood-cell sequencing when performed, and standardized reporting language such as “ctDNA not detected” rather than “ctDNA negative” [5,11,12,40].

## 6. ctDNA for Baseline Risk, Treatment Response, MRD, and Molecular Relapse

The most clinically developed ctDNA applications in sarcoma include baseline risk enrichment, treatment-response monitoring, postoperative MRD detection, and molecular relapse surveillance. These applications are related but not identical. Baseline ctDNA detection often reflects tumor burden and aggressive biology; on-treatment ctDNA dynamics may indicate response or resistance; postoperative ctDNA detection may identify residual molecular disease; and later reappearance or rise in ctDNA during follow-up may indicate molecular relapse.

At baseline, detectable ctDNA has been associated with adverse disease features in several sarcoma settings. In Children’s Oncology Group analyses, ctDNA was detectable in more than half of newly diagnosed Ewing sarcoma and osteosarcoma cases, and detectable ctDNA was associated with inferior outcomes, particularly in localized Ewing sarcoma [31]. In high-grade osteosarcoma, lpWGS-based ctDNA quantification from the OS2006 trial improved outcome estimation when combined with clinical parameters [17]. These findings suggest that baseline ctDNA may function as a risk-enrichment biomarker, although performance depends on assay type, disease burden, and biological shedding.

During treatment, the most consistent observation is that falling ctDNA levels tend to parallel response, whereas persistent or rising ctDNA suggests resistant or progressive disease [13,16,22,30]. In translocation-associated sarcomas, breakpoint-guided assays provide a direct quantitative marker of tumor-specific DNA. In osteosarcoma, longitudinal tumor-informed ctDNA detection can reflect response to adjuvant chemotherapy, with clearance associated with favorable disease course in selected patients and persistent detection suggesting MRD or resistance [16]. These observations support response-adaptive monitoring, but they do not yet establish that ctDNA-guided treatment changes improve outcomes.

MRD detection is the most clinically ambitious use of ctDNA in sarcoma. It requires substantially greater analytical sensitivity than baseline genotyping because tumor-derived DNA may be present at very low levels after surgery or definitive therapy [5,9,10,11,12]. Osteosarcoma currently provides the strongest proof-of-principle among complex-karyotype sarcomas. In the largest osteosarcoma MRD cohort to date, tumor-informed MRD panels were successfully generated in 71 of 83 patients, and 59 patients had customized panels with available blood samples [16]. Postoperative ctDNA detection predicted inferior event-free survival, and ctDNA preceded positive imaging in five patients, with a mean lead time of 92.6 days [16]. These data support the prognostic relevance of tumor-informed ctDNA monitoring in osteosarcoma.

The osteosarcoma experience also clarifies why tumor-informed approaches are valuable. Marked mutational heterogeneity and a high proportion of nonrecurrent mutations make a uniform tumor-agnostic MRD panel less attractive [16]. Tumor-informed assays can track multiple patient-specific alterations at high depth, improving sensitivity for low-level residual disease [9,16]. However, they may miss newly acquired mutations or subclonal evolution not represented in the original tumor panel, and they require adequate tissue and robust assay design.

Translocation-associated sarcomas offer a complementary MRD model. In Ewing sarcoma and other breakpoint-defined tumors, the disease-defining structural variant provides a stable and highly specific target for longitudinal monitoring [13,22,23]. Long-term clinical use in translocation-associated sarcomas has shown that ctDNA levels correlate with disease course and are higher in progressive disease than in remission [22]. However, unilocular or low-volume recurrence may remain undetected, emphasizing that ctDNA should not replace imaging [22].

Additional subtype-specific evidence extends the MRD concept. In myxoid liposarcoma, plasma detection of structural breakpoints and additional variants can reflect disease course and improve quantitative monitoring [15,24,35]. In chondrosarcoma, IDH1/2- or GNAS-mutant ctDNA detection has been proposed for diagnosis, risk stratification, and residual disease assessment in molecularly informative tumors [18,20]. In chordoma, mutation-based and methylation-based plasma approaches suggest potential for monitoring and prognostication, although evidence remains early and less standardized [19,38].

Current evidence supports a strong but measured conclusion: ctDNA can provide clinically meaningful prognostic and response information in selected sarcoma subtypes, but routine treatment modification based solely on ctDNA remains premature. The appropriate near-term role is risk stratification, clinical-trial enrichment, closer imaging follow-up, and multidisciplinary reassessment when ctDNA findings and clinical context suggest increased relapse risk. Representative original studies supporting these subtype-specific liquid-biopsy applications are summarized in Table 1. Overall, evidence maturity differs substantially across sarcoma subtypes and liquid-biopsy applications. Tumor-informed ctDNA monitoring in osteosarcoma and breakpoint-guided ctDNA monitoring in translocation-associated sarcomas currently have the clearest longitudinal clinical support, although both still require prospective multicenter validation before routine treatment decisions can be based on ctDNA alone [13,16,17,22,23,24]. In contrast, broad plasma panels, CNA-based WGS, methylation-based assays, cfRNA approaches, and CTC-based strategies remain more exploratory or proof-of-concept in most sarcoma subtypes, particularly when applied to low-volume MRD or routine surveillance settings [25,26,27,28,29,30,31,32,33,34,35,36,37,38,39].

## 7. Subtype-Specific Evidence Summary

The current evidence supports a subtype-specific and clinical-question-matched approach to liquid biopsy in sarcoma rather than a single universal platform. This distinction is essential because the most informative circulating signal differs according to sarcoma biology. Fusion-driven tumors are generally suited to breakpoint- or fusion-aware assays, whereas complex-karyotype tumors may require tumor-informed sequencing, copy-number-based approaches, methylation-based strategies, or complementary CTC analysis.

Osteosarcoma represents one of the most developed models for tumor-informed ctDNA monitoring among complex-karyotype sarcomas. In this setting, patient-specific ctDNA assays can support postoperative MRD assessment, relapse-risk stratification, and longitudinal response monitoring [16,17]. However, osteosarcoma remains biologically heterogeneous, and ctDNA not detected in plasma should not be interpreted as definitive absence of disease.

Ewing sarcoma and other translocation-associated sarcomas provide a different model because disease-defining structural variants can serve as highly specific molecular targets for longitudinal monitoring [13,22,23]. Synovial sarcoma and myxoid liposarcoma similarly illustrate the value of structural-variant or breakpoint-informed approaches, although available cohorts remain limited and assay standardization is still needed [14,15,24,35]. In chondrosarcoma and chordoma, mutation- and methylation-based strategies may be informative in selected molecularly defined cases, but these approaches are not universally applicable across all patients [18,19,20,38].

CTC-based methods provide complementary biological information, particularly in osteosarcoma, but their broader application across soft-tissue sarcomas remains limited by platform heterogeneity and uncertain prevalence [25,26,27,28,29,39]. For broader complex-karyotype soft-tissue sarcomas, the evidence remains more exploratory. Commercial or large-panel plasma profiling may identify clinically relevant alterations in selected advanced cases, but variable tissue–plasma concordance, low tumor fraction, and inconsistent copy-number detection limit its use as a general surveillance strategy [32,33,34,35,36].

## 8. Circulating Tumor Cells as a Complementary Biomarker

Compared with ctDNA, the CTC literature in sarcoma is less standardized but remains clinically informative. CTCs represent intact tumor cells rather than fragmented nucleic acids, allowing assessment of cellular phenotype, metastatic competence, epithelial–mesenchymal plasticity, and potentially treatment-resistant subpopulations [25,26,27,28,29]. This is biologically relevant in sarcoma because hematogenous dissemination is central to metastatic progression. However, CTC detection is technically difficult in mesenchymal tumors, especially when platforms rely on epithelial markers such as EpCAM or cytokeratins. Sarcoma CTC assays therefore require size-based, mesenchymal-marker, or metabolism-based enrichment strategies. Several technical limitations are particularly relevant to CTC detection in mesenchymal tumors. Unlike epithelial malignancies, many sarcomas do not consistently express epithelial capture markers such as EpCAM or cytokeratins, which reduces the sensitivity of conventional epithelial-marker-based CTC platforms. Size-based enrichment may improve capture of larger tumor cells, but it can also be affected by overlap between tumor cells and activated leukocytes, tumor-cell deformability, and platform-specific filtration thresholds. Mesenchymal-marker or metabolism-based approaches may be more biologically appropriate for sarcoma, but they remain less standardized and may vary according to tumor subtype, treatment exposure, CTC phenotype, and sample-processing conditions [25,26,27,28,29,39]. These technical issues explain why CTC data in sarcoma should currently be interpreted as complementary and exploratory rather than as a standardized clinical surveillance tool.

Osteosarcoma currently provides the strongest CTC evidence base in sarcoma. In one study, CTCs were detected in 30 of 32 osteosarcoma patients before treatment, with a mean pretreatment CTC count of 14.06 ± 9.08 and no CTCs detected in 10 healthy volunteers [27]. Another osteosarcoma study detected baseline CTCs in 28 of 30 patients, with a median of 6 CTCs per 5 mL of blood [25]. Dai et al. detected CTCs in 43 of 50 osteosarcoma patients, including all 16 patients with metastatic disease and 27 of 34 patients without metastasis, and reported that IMP3-positive CTC count showed an AUC of 0.873 for identifying metastasis, with 87.5% sensitivity and 82.4% specificity [26]. These data suggest that CTCs may be detectable in a substantial proportion of osteosarcoma patients when mesenchymal-compatible platforms are used.

CTC phenotype may also provide information beyond enumeration alone. Mesenchymal and hybrid epithelial/mesenchymal CTC phenotypes have been associated with advanced stage, metastatic disease, and treatment response in osteosarcoma [25,26,27]. A hexokinase 2-based CTC platform developed for osteosarcoma further supports the potential of tumor-biology-adapted CTC detection, showing concordance with therapy response and disease-free survival in both serial and single-time-point analyses [28]. These findings indicate that CTCs may capture disseminating tumor-cell biology not fully reflected by ctDNA.

However, osteosarcoma findings should not be generalized across all sarcomas. Evidence in soft-tissue sarcoma remains more limited. The CIRCUS study showed that CTCs could be isolated from peripheral blood in 13 soft-tissue sarcoma patients, with heterogeneous epithelial and mesenchymal marker expression, but did not establish a definitive prevalence threshold or clinical decision framework [29]. Surgical salvaged-blood studies in osteosarcoma further support the feasibility of CTC detection, but they should be interpreted as pilot data rather than as evidence for routine clinical surveillance [39]. Therefore, while CTCs are promising, especially in osteosarcoma, their broader role across soft-tissue sarcoma remains exploratory.

At present, CTCs should be viewed as complementary rather than competing biomarkers. ctDNA remains more mature for subtype-specific molecular monitoring, MRD detection, and molecular relapse surveillance. CTCs may add value when the clinical question involves metastatic potential, cellular phenotype, treatment-resistant subpopulations, or translational biology. Their routine clinical implementation will require standardized enrichment methods, reproducible detection thresholds, and prospective validation across sarcoma subtypes. The quantitative context for ctDNA and CTC performance across representative sarcoma studies is summarized in Table 2.

## 9. Integration with Imaging and Clinical Workflows

The most clinically realistic role of liquid biopsy in sarcoma is not to replace imaging, but to support imaging interpretation and refine surveillance intensity. Current guidelines continue to rely on radiologic assessment, particularly chest imaging for metastatic surveillance and MRI or site-specific imaging for local disease control [1,2,6,7,8]. Liquid biopsy should therefore be incorporated as a decision-support tool within established imaging-based follow-up rather than as a standalone surveillance pathway.

A practical integration model includes four main scenarios. First, if ctDNA is not detected and imaging is stable, standard surveillance should continue. This finding may be reassuring, especially if ctDNA was previously detectable, but it should not be interpreted as definitive absence of disease because low-volume or anatomically localized recurrence may remain below the plasma detection threshold [5,22]. Second, if ctDNA is detected or rising while imaging is negative or equivocal, the result should prompt repeat plasma testing, confirmation of assay validity, multidisciplinary review, and consideration of intensified or targeted imaging. Depending on subtype and recurrence pattern, this may include shorter-interval CT, targeted MRI, PET/CT, or whole-body imaging.

Third, if ctDNA declines during neoadjuvant or systemic therapy, it may support biological response, especially when radiographic size change is delayed or modest. Conversely, persistent or rising ctDNA during therapy may suggest resistant disease and justify earlier restaging or multidisciplinary reassessment [13,16,22,30]. Fourth, postoperative ctDNA detection may identify a high-risk state even when imaging shows no measurable disease. In this setting, ctDNA should currently guide risk discussion, clinical-trial consideration, and surveillance intensity rather than automatic treatment escalation outside evidence-based protocols.

This integrative model is particularly relevant for high-risk osteosarcoma and translocation-associated sarcomas. In osteosarcoma, postoperative tumor-informed ctDNA may identify patients at increased relapse risk despite apparent remission [16]. In Ewing sarcoma and other breakpoint-defined sarcomas, serial ctDNA measurements can provide a highly specific adjunctive marker during remission and follow-up [13,22,23]. However, localized or unilocular recurrence may remain undetectable by ctDNA, reinforcing the need for continued imaging [22].

Implementation should occur within specialized sarcoma care pathways. Liquid-biopsy results should be interpreted alongside tumor subtype, disease stage, treatment history, prior ctDNA detectability, imaging findings, pathology, and pretest probability of recurrence. This is especially important in rare tumors, where false reassurance from “not detected” results and overinterpretation of low-level positivity may both have clinical consequences. Until prospective interventional trials demonstrate outcome benefit, liquid biopsy should enhance, not replace, imaging-based surveillance and multidisciplinary clinical judgment.

## 10. Limitations and Quality of Current Evidence

The current evidence base for liquid biopsy in sarcoma is encouraging but remains insufficient for universal routine implementation. First, most studies are limited by small cohort sizes, single-center designs, heterogeneous histologic inclusion, and variable sampling schedules [4,16,22,32,33,34,35,36]. Even in settings with strong preliminary data, such as osteosarcoma MRD monitoring and translocation-associated sarcoma surveillance, independent validation across larger multicenter cohorts is required [16,22].

A structured interpretation of the current evidence also requires attention to study quality. Most available sarcoma liquid-biopsy studies should be considered hypothesis-generating rather than practice-changing because they are frequently retrospective, single-center, and limited by small sample sizes. Several cohorts combine biologically distinct sarcoma subtypes, disease stages, treatment contexts, and sampling time points, which limits statistical power and complicates subtype-specific interpretation. Selection bias may also occur when studies preferentially include patients with available tumor tissue, sufficient plasma volume, higher tumor burden, metastatic disease, or technically successful assays. These factors may overestimate detection rates and underestimate the frequency of non-informative or “ctDNA not detected” results in real-world surveillance.

Assay-related bias is another major limitation. Studies differ in plasma-processing protocols, cfDNA input, sequencing depth, error suppression, tumor-informed versus tumor-naïve design, positivity thresholds, and handling of clonal hematopoiesis. These differences make direct comparison of sensitivity, specificity, lead time, and prognostic performance difficult across studies. Statistical limitations are also common, including small event numbers, limited multivariable adjustment, inconsistent reporting of confidence intervals, and incomplete external validation. Finally, clinical endpoints remain heterogeneous, with some studies using radiographic progression and others using event-free survival, disease-free survival, molecular relapse, imaging lead time, or treatment response. Future studies should therefore define standardized clinical endpoints before enrollment and report assay performance according to tumor subtype, disease stage, treatment setting, sampling time point, and imaging comparator.

Second, assay heterogeneity limits comparison across studies. Published work differs in plasma-processing protocols, cfDNA input, sequencing depth, error suppression, assay type, positivity thresholds, timing of blood collection, and correlation with imaging or clinical endpoints [11,12,40]. This heterogeneity is particularly consequential in sarcoma because tumor-derived signal may be low and subtype-specific. Future studies should report pre-analytical and analytical variables transparently, including plasma volume, tube type, processing delay, cfDNA input, limit of detection, limit of quantification, tumor fraction when available, and handling of clonal hematopoiesis [5,11,12,40].

Third, low-shedding disease remains a fundamental limitation. Sarcomas do not consistently release detectable ctDNA into plasma, and “not detected” results may reflect low tumor burden, localized disease, limited vascular shedding, anatomical site, treatment effect, or insufficient assay sensitivity rather than true absence of disease [5,22]. This limitation is most important in postoperative MRD and molecular relapse settings, where the expected tumor-derived fraction is extremely low.

Additional sarcoma liquid-biopsy studies, including osteosarcoma ctDNA, bone sarcoma cfDNA, cfRNA fusion-gene analysis, chordoma monitoring, non-metastatic soft-tissue sarcoma profiling, structural-variant detection, and personalized sarcoma ctDNA monitoring cohorts, remain exploratory and require prospective validation before clinical implementation [41,42,43,44,45,46,47]. Implementation should also occur within specialized sarcoma care pathways and established multidisciplinary surveillance frameworks [48].

Fourth, clinical utility continues to lag behind clinical validity. Multiple studies show that ctDNA detection after treatment is prognostic and may precede imaging relapse in selected cases [16,22,24]. However, it remains unclear whether acting on these findings improves survival, preserves function, or reduces treatment toxicity. Prospective interventional trials are therefore needed to test whether ctDNA-guided strategies such as intensified imaging, earlier salvage evaluation, adaptive therapy, or trial enrollment improve patient outcomes.

CTC research has additional barriers. Detection platforms differ widely, sarcoma-specific CTC markers are not standardized, and most clinically informative data are concentrated in osteosarcoma [25,26,27,28,29,39]. Future studies should define reproducible detection thresholds, clarify whether CTC phenotype adds independent prognostic value beyond ctDNA and imaging, and test whether CTC-based monitoring is scalable across soft-tissue sarcoma subtypes.

## 11. Implementation Challenges and Future Research Priorities

Implementation of liquid biopsy in sarcoma should occur within specialized sarcoma care pathways and established multidisciplinary surveillance frameworks [48]. The contemporary literature reinforces that ctDNA technologies are clinically promising but remain early in sarcoma, with ongoing barriers related to assay selection, disease burden, low tumor fraction, pre-analytical variability, and clinical validation [49]. In rare mesenchymal malignancies such as adult rhabdomyosarcoma, detailed molecular characterization also illustrates why subtype-specific biology must be understood before biomarker-driven monitoring strategies can be generalized across sarcoma [50]. Broader molecular-methods reviews in soft-tissue sarcoma similarly highlight that liquid biopsy should be integrated within a wider diagnostic and theragnostic framework that includes tissue-based genomics, transcriptomics, methylation profiling, and standardized molecular testing [51].

Future research should prioritize prospective multicenter studies with harmonized sampling schedules, subtype-specific assay selection, standardized reporting, and clinically meaningful endpoints. Prospective trial designs should distinguish between observational validation and interventional utility. Observational studies should test whether ctDNA or CTC dynamics independently predict relapse after adjustment for stage, histology, treatment response, surgical margins, and imaging findings. Interventional trials should then test whether predefined biomarker-triggered actions, such as intensified imaging, earlier multidisciplinary review, adaptive systemic therapy, or clinical-trial enrollment, improve event-free survival, overall survival, limb preservation, functional outcomes, or patient-reported quality of life. Harmonized endpoints should include radiographic recurrence, molecular relapse, lead time over imaging, event-free survival, disease-free survival, overall survival, assay failure rate, false-positive rate, false-negative rate, turnaround time, cost, and clinical actionability.

Future surveillance research should also evaluate integration of liquid biopsy with quantitative imaging, radiomics, and artificial intelligence. Radiomics and AI-based imaging models are increasingly being explored in bone and soft-tissue tumors for diagnosis, grading, prognosis, treatment-response prediction, and recurrence-risk assessment, but reproducibility, segmentation variability, external validation, interpretability, and clinical deployment remain major barriers [52]. A future liquid-biopsy-guided surveillance model could combine ctDNA kinetics, CTC phenotype, imaging features, radiomic signatures, and clinicopathologic variables to generate multimodal risk scores. However, such models should be developed cautiously using multicenter datasets, predefined endpoints, explainable algorithms, external validation cohorts, and prospective testing before clinical adoption. At present, radiomics–liquid biopsy integration should be viewed as a research priority rather than an established surveillance strategy [53].

Additional translational literature further supports the need for biologically grounded implementation. Integrated genomic and pharmacologic profiling in extremity sarcomas illustrates how molecular characterization may help define treatment-response biology and future predictive biomarker strategies [54]. Studies of soft-tissue sarcoma immunity also emphasize that genomic architecture, immune microenvironment, and therapeutic responsiveness are interrelated, supporting the need to interpret circulating biomarkers within broader tumor-biological context [55]. Finally, clinical-practice reviews of sarcoma liquid biopsy highlight that although ctDNA and CTC approaches can provide real-time information on tumor genetics and disease state, technical and clinical implementation barriers remain substantial [56]. A practical subtype-level summary of liquid-biopsy implementation, including suitable assay approach, best-supported clinical use, evidence maturity, and main limitation, is provided in Table 3.

## 12. Conclusions

Liquid biopsy is reshaping how disease activity is understood in bone and soft-tissue sarcomas, but its clinical value depends on matching the assay to tumor biology and clinical purpose. The most defensible framework is therefore genomics-guided rather than platform-driven. This approach recognizes that translocation-associated sarcomas, complex-karyotype tumors, mutation-defined subtypes, and methylation-informative tumors require different liquid-biopsy strategies.

Among available analytes, ctDNA has the greatest near-term clinical relevance. Its best-supported current applications include baseline risk assessment, treatment-response monitoring, postoperative MRD detection, and molecular relapse surveillance, particularly in osteosarcoma and translocation-associated sarcomas. CTCs provide complementary cellular and prognostic information, especially in osteosarcoma, but currently remain less standardized and less mature than ctDNA for routine surveillance.

The field has moved beyond simple proof of detectability. The next priority is to establish how liquid-biopsy results should be interpreted, when they should trigger further imaging or intervention, and whether biomarker-guided decisions improve patient outcomes. Until such evidence is available, liquid biopsy should be integrated cautiously as an adjunct to imaging, specialist pathology, molecular diagnostics, and multidisciplinary sarcoma care. Its greatest current value lies not in replacing scans, but in adding molecular context to them.

## Figures and Tables

**Table 1:** Representative original studies evaluating liquid-biopsy biomarkers in bone and soft-tissue sarcomas. The table summarizes clinically informative sarcoma studies according to subtype, cohort, biomarker, assay platform, clinical application, and key findings.

Author/Year	Sarcoma Subtype	Study Design/Cohort	Biomarker	Platform/Assay	Main Clinical Application	Key Findings
Fu et al., 2024, [16]	Osteosarcoma	Prospective longitudinal cohort; WES performed in 83 patients; tumor-informed MRD panels generated in 71/83; plasma analysis available in 59 patients	ctDNA	Tumor-informed multiplex PCR-based deep NGS after tumor WES	Postoperative MRD and relapse prediction	Postoperative ctDNA positivity was associated with worse EFS; ctDNA preceded imaging relapse in five patients, with mean lead time of 92.6 days
Audinot et al., 2024, [17]	High-grade osteosarcoma	Translational study from the prospective OS2006 trial; 183 patients evaluated for diagnosis ctDNA; 465 plasma samples across diagnosis, pre-surgery, and end of treatment	ctDNA	Low-pass whole-genome sequencing/copy-number alteration-based ctDNA quantification	Baseline risk stratification and outcome prediction	Diagnosis ctDNA quantification was an independent prognostic factor and improved prognostic stratification when added to metastatic status or histologic response
Shah et al., 2021, [30]	Pediatric sarcomas, including Ewing sarcoma and other translocation/CNA-driven tumors	Prospective plasma cohort; 17 patients, 64 plasma samples	ctDNA	CAPP-Seq for translocations plus off-target CNA analysis using ichorCNA	Molecular detection, response monitoring, and relapse detection	Pretreatment translocations detected in 13 patients and confirmed by tumor sequencing in 12; CNAs detected in 7 patients; ctDNA correlated with metastatic status and clinical response
Shulman et al., 2018, [31]	Ewing sarcoma and osteosarcoma	Children’s Oncology Group correlative study; 94 Ewing sarcoma and 72 osteosarcoma patients	ctDNA	Hybrid-capture NGS for Ewing sarcoma; ultra-low-pass WGS for osteosarcoma	Baseline prognostication	ctDNA was detectable in 53% of newly diagnosed Ewing sarcoma and 57% of osteosarcoma cases; detectable ctDNA was associated with inferior outcomes, especially in localized Ewing sarcoma
Joch et al., 2025, [22]	Translocation-associated sarcomas	Retrospective longitudinal cohort; 34 patients, 285 ctDNA samples; median follow-up 97 weeks	ctDNA	Translocation-specific ctDNA quantification	Long-term MRD monitoring and surveillance	ctDNA levels were significantly associated with clinical course; ctDNA was higher in multilocular recurrence but could remain negative in some unilocular recurrences
Eisenhardt et al., 2022, [14]	Synovial sarcoma	Translational cohort; tumor profiling in 25 SS patients; plasma analysis in 29 samples from 15 patients	ctDNA	Subtype-specific targeted NGS for SS18–SSX breakpoints plus patient-specific exome-informed panels	Tumor dynamics and recurrence monitoring	Breakpoint ctDNA detected in plasma with 40% sensitivity and 100% specificity; patient-specific panels improved sensitivity and longitudinal monitoring
Schmid et al., 2025, [15]	Myxoid liposarcoma, with validation including one synovial sarcoma case	Analytical-clinical validation; dilution series using tumor DNA from 11 MLS patients and one cell line; plasma validation in 36 samples from 2 MLS patients and 1 SS patient	ctDNA	Combined structural-variant breakpoint and SNV tracking with UMI-supported NGS	Improved ctDNA quantification and assay optimization	UMI correction reduced false-positive SNV calls; additional filters improved specificity without major sensitivity loss; plasma validation supported clinical-course correlation
Lyskjær et al., 2021, [18]	Central chondrosarcoma with IDH1/2 or GNAS mutations	Multi-institutional cohort; 145 cartilaginous tumors recruited; ctDNA assessed in 83 molecularly informative patients	ctDNA	Mutation-specific ddPCR for IDH1/2 and GNAS	Risk stratification, prognosis, and postoperative detection	Preoperative ctDNA detected in 31/83 patients; postoperative ctDNA detected in 12/31; ctDNA associated with high-grade disease and poor prognosis
Tsoi et al., 2021, [36]	Mixed bone and soft-tissue sarcomas	Pilot cohort; 64 patients, 70 plasma samples; ctDNA tested in 6 tumor-informed cases	cfDNA/ctDNA	cfDNA quantification plus tumor-informed ddPCR	Pan-sarcoma feasibility and prognostic signal	cfDNA detected in 69/70 samples; lower cfDNA was associated with better 1-year DFS; tumor-specific ctDNA detected in 5/6 tested cases
Mu et al., 2022, [28]	Osteosarcoma	CTC translational study; 12-patient training cohort and 8-patient validation cohort	CTCs	Hexokinase 2-based CTC detection and surveillance platform	Therapy response and prognosis prediction	Dynamic CTC model showed 92% consistency with clinical outcomes; validation single CTC test showed 100% consistency with therapy response and 87.5% with DFS

**Table 2:** Quantitative performance benchmarks for interpreting liquid-biopsy evidence in sarcoma.

Clinical Context	Key Evidence Source	Quantitative Finding	Interpretation for This Review
Broader solid-tumor MRD benchmark	ESMO ctDNA recommendations and solid-tumor MRD reviews [5,9,10]	ctDNA MRD/molecular-relapse detection generally shows high relapse-prediction specificity, but early post-treatment sensitivity is often limited.	Sarcoma false-negative results should be interpreted within the broader MRD field; low sensitivity in low-burden disease is not unique to sarcoma.
Assay-validation and reporting context	BloodPAC and AMP/CAP recommendations [11,12]	ctDNA assays require clear reporting of analytical performance, pre-analytical handling, and assay-specific limitations.	Sarcoma studies should report assay sensitivity, specificity, cfDNA input, processing variables, and interpretation limits.
Osteosarcoma tumor-informed MRD	Fu et al. [16]	Tumor-informed MRD panels were generated in 71/83 patients; plasma analysis was available in 59 patients; ctDNA preceded imaging relapse in 5 patients, with a mean lead time of 92.6 days.	Strong current sarcoma example supporting tumor-informed postoperative MRD and molecular-relapse surveillance.
Osteosarcoma baseline risk stratification	Audinot et al. [17]	OS2006 included 183 patients evaluated for diagnostic ctDNA and 465 plasma samples; ctDNA quantification improved outcome estimation.	Baseline ctDNA can enrich risk assessment, but this is distinct from ultra-sensitive MRD detection.
Translocation-associated sarcoma monitoring	Joch et al. [22]	Retrospective cohort of 34 patients and 285 ctDNA samples; median follow-up was 97 weeks; ctDNA levels were associated with clinical disease course.	Breakpoint-guided ctDNA monitoring is clinically informative, but localized or unilocular recurrence may remain undetected.
Osteosarcoma CTC prevalence	Li et al., Dai et al., Wu et al., and Mu et al. [25,26,27,28]	CTCs were detected in 28/30, 43/50, and 30/32 osteosarcoma patients across three studies; IMP3-positive CTCs showed AUC 0.873 for metastasis in one cohort.	CTC detection appears relatively frequently in osteosarcoma when sarcoma-adapted platforms are used, but these findings should not be generalized to all sarcomas.
Soft-tissue sarcoma CTC feasibility	Young et al. [29]	CTC isolation was feasible in a small soft-tissue sarcoma pilot cohort, but no definitive clinical decision threshold was established.	STS CTC work remains exploratory and less mature than osteosarcoma CTC evidence or ctDNA-based monitoring.
Pediatric sarcoma structural-variant and CNA detection	Shah et al. [30]	Translocations were detected in pretreatment plasma from 13 patients and confirmed by tumor sequencing in 12; CNAs were detected in 7 patients.	A combined structural-variant and CNA strategy is feasible in pediatric sarcomas and supports subtype-matched assay design.
Ewing sarcoma and osteosarcoma baseline detection	Shulman et al. [31]	ctDNA was detectable in 53% of newly diagnosed Ewing sarcoma and 57% of osteosarcoma cases.	Detection at diagnosis is feasible in many, but not all, bone sarcoma patients; sensitivity depends on tumor burden, assay design, and shedding.
Commercial/broad ctDNA profiling in advanced STS	Demoret et al. [32]	ctDNA profiling identified alterations in selected advanced STS cases, but concordance with tissue varied by subtype and alteration type.	Broad plasma panels may help selected advanced cases but are not yet reliable as general sarcoma surveillance tools.
CNA-based liquid biopsy in sarcoma	Anderson et al. [33]	LP-WGS detected ctDNA in 9/13 sarcoma plasma samples; positive samples had ctDNA fractions of 6–11% and plasma ctDNA concentrations of 0.04–5.6 ng/mL.	CNA-based assays are relevant for complex-karyotype sarcomas but should not be presented as universally sensitive MRD tools.
Advanced STS precision profiling	Blanchi et al. [34]	Large-panel ctDNA sequencing was evaluated in advanced STS and may identify therapeutically relevant alterations in selected patients.	Advanced-disease profiling should be distinguished from MRD surveillance.
Myxoid liposarcoma ctDNA refinement	Eisenhardt et al. [35]	Targeted next-generation sequencing of circulating free DNA enabled non-invasive tumor detection in myxoid liposarcoma.	Even within fusion-driven tumors, assay optimization may improve monitoring accuracy.
Mixed bone and soft-tissue sarcoma feasibility	Tsoi et al. [36]	cfDNA was detected in 69/70 samples; tumor-specific ctDNA was detected in 5/6 tumor-informed cases.	Pan-sarcoma feasibility exists, but evidence remains preliminary and requires subtype-specific validation.
Plasma tumor DNA versus RNA in Ewing sarcoma	Bodlak et al. [37]	Plasma tumor DNA was more reproducible than plasma tumor RNA for Ewing sarcoma monitoring.	cfRNA is promising but should not be presented as equivalent to ctDNA for routine monitoring.
Chordoma mutation-based detection	Mattox et al. [38]	ctDNA mutations were detected in chordoma patients and may correlate with clinical disease status.	Chordoma supports selected mutation-based monitoring but requires further validation.
Surgical osteosarcoma CTC pilot	Nawan et al. [39]	CTCs were detected in salvaged blood in a pilot osteosarcoma surgical cohort.	Supports biological feasibility but should not be used as evidence for ctDNA assay validation or consensus reporting standards.

Abbreviations: AUC, area under the curve; CNA, copy-number alteration; CTC, circulating tumor cell; ctDNA, circulating tumor DNA; LP-WGS, low-pass whole-genome sequencing; MRD, minimal residual disease; STS, soft-tissue sarcoma.

**Table 3:** Subtype-level clinical implementation summary for liquid biopsy in bone and soft-tissue sarcomas.

Sarcoma Subtype/Group	Most Suitable Current Assay Approach	Best-Supported Clinical Use	Evidence Maturity	Main Limitation
Osteosarcoma	Tumor-informed ctDNA; CNA-based WGS; CTCs as adjunct	Postoperative MRD, relapse-risk stratification, treatment-response monitoring	Moderate and growing	Genomic heterogeneity, low-shedding disease, tissue requirement for tumor-informed assays
Ewing sarcoma	Breakpoint-guided ddPCR or fusion-aware NGS	Baseline risk assessment, response monitoring, molecular relapse surveillance	Moderate	Patient-specific breakpoint design; low-volume recurrence may remain undetected
Synovial sarcoma	SS18::SSX fusion-aware sequencing or breakpoint-guided assay	Longitudinal monitoring in selected patients	Early to moderate	Small cohorts; limited assay standardization
Myxoid liposarcoma	Fusion/breakpoint-informed ctDNA with selected SNV tracking	Disease-course monitoring and recurrence assessment	Early to moderate	Very low tumor fraction in some samples
Chondrosarcoma	IDH1/2 or GNAS mutation-specific ddPCR or targeted NGS	Molecular diagnosis support, risk stratification, residual disease assessment in mutation-positive tumors	Early to moderate	Only applicable to molecularly informative cases
Chordoma	Tumor-informed mutation tracking; methylation-based profiling	Monitoring and prognostic classification	Early	Rare disease; limited longitudinal validation
Broader complex-karyotype STS	Tumor-informed NGS, CNA-based WGS, or broad plasma profiling	Advanced disease profiling, exploratory response monitoring, selected surveillance contexts	Early	Variable tissue–plasma concordance, low tumor fraction, no universal biomarker
CTC-based approaches	Size-based, mesenchymal-marker, or metabolism-based enrichment	Prognostic and biological insight, especially in osteosarcoma	Early to moderate in osteosarcoma; early in STS	Platform heterogeneity, lack of standardized markers, unclear clinical thresholds

Abbreviations: CNA, copy-number alteration; CTC, circulating tumor cell; ctDNA, circulating tumor DNA; ddPCR, digital droplet PCR; MRD, minimal residual disease; NGS, next-generation sequencing; SNV, single-nucleotide variant; STS, soft-tissue sarcoma; WGS, whole-genome sequencing.

## Data Availability

No new data were created or analyzed in this study. Data sharing is not applicable to this article.

## Abbreviations

ACR	American College of Radiology
CAPP-Seq	Cancer Personalized Profiling by Deep Sequencing
cfDNA	cell-free DNA
cfRNA	cell-free RNA
CNA	copy-number alteration
CNAs	copy-number alterations
COG	Children’s Oncology Group
CTCs	circulating tumor cells
ctDNA	circulating tumor DNA
DDLPS	dedifferentiated liposarcoma
ddPCR	digital droplet polymerase chain reaction
ESMO	European Society for Medical Oncology
EURACAN	European Reference Network for Rare Adult Solid Cancers
GENTURIS	European Reference Network on Genetic Tumor Risk Syndromes
IDH	isocitrate dehydrogenase
LMS	leiomyosarcoma
LPS	liposarcoma
MRI	magnetic resonance imaging
MRD	minimal residual disease
NGS	next-generation sequencing
PET-CT	positron emission tomography–computed tomography
STS	soft-tissue sarcoma
UPS	undifferentiated pleomorphic sarcoma
UMI	unique molecular identifier
